# Supplementary material for: A Humanized Yeast Phenomic Model of Deoxycytidine Kinase to Predict Genetic Buffering of Nucleoside Analog Cytotoxicity
Source: Genes (Basel). 2019 Sep 30;10(10):770. doi: 10.3390/genes10100770 (PMC6826991; doi:10.3390/genes10100770)
Supplement: Supplementary file 1 [file genes-10-00770-s001.zip › Additional_Files/Additional_File7_GOTermHeatmaps.zip!/Additional_File7_GOTermHeatmaps/ribonucleoprotein_complex_binding.pdf]

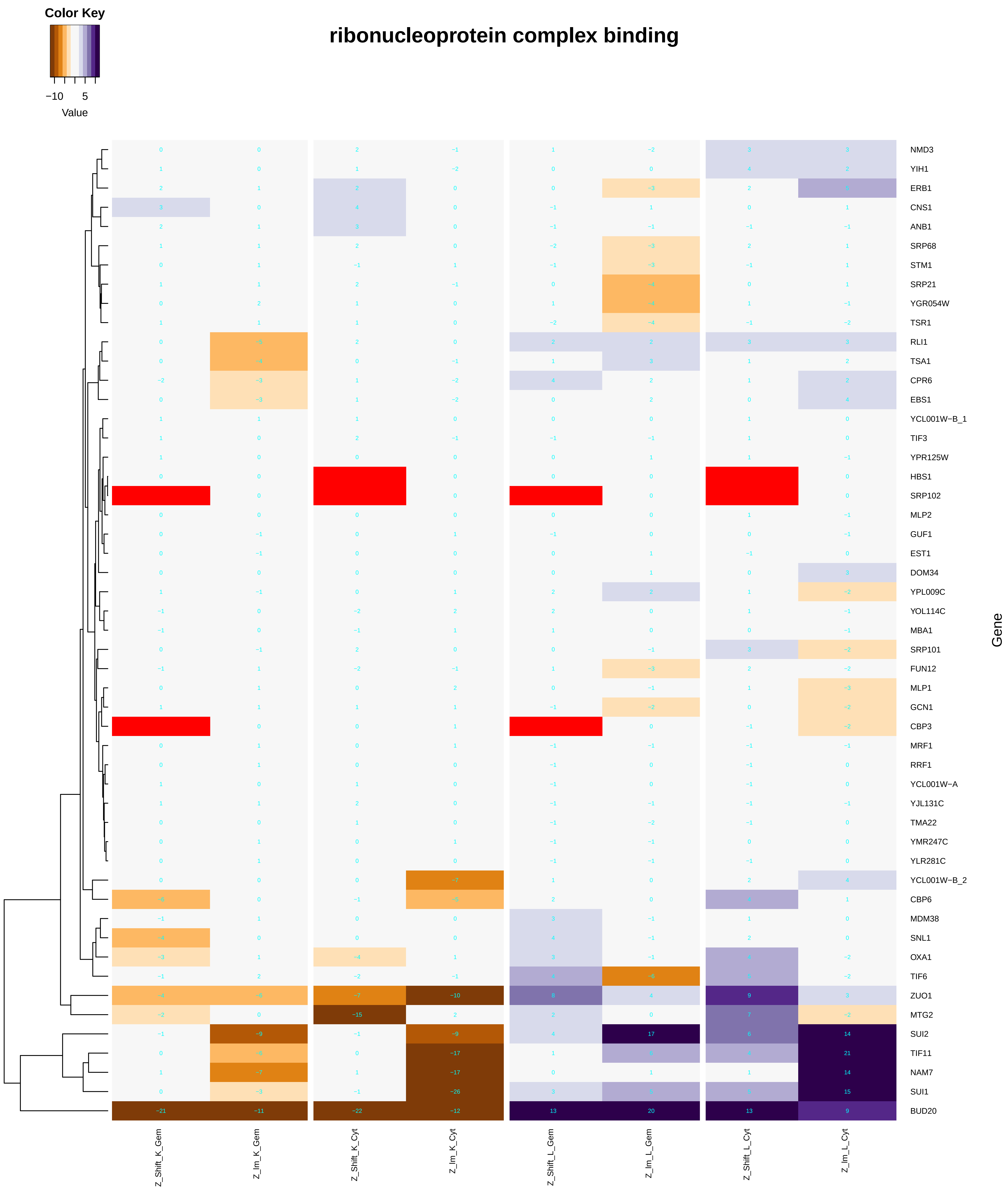

Color Key
−10
5
Value
ribonucleoprotein complex binding
NMD3
YIH1
ERB1
CNS1
ANB1
SRP68
STM1
SRP21
YGR054W
TSR1
RLI1
TSA1
CPR6
EBS1
YCL001W−B_1
TIF3
YPR125W
HBS1
SRP102
MLP2
GUF1
EST1
DOM34
YPL009C
YOL114C
MBA1
SRP101
FUN12
MLP1
GCN1
CBP3
MRF1
RRF1
YCL001W−A
YJL131C
TMA22
YMR247C
YLR281C
YCL001W−B_2
CBP6
MDM38
SNL1
OXA1
TIF6
ZUO1
MTG2
SUI2
TIF11
NAM7
SUI1
BUD20
Gene
Z_Shift_K_Gem
Z_Im_K_Gem
Z_Shift_K_Cyt
Z_Im_K_Cyt
Z_Shift_L_Gem
Z_Im_L_Gem
Z_Shift_L_Cyt
Z_Im_L_Cyt

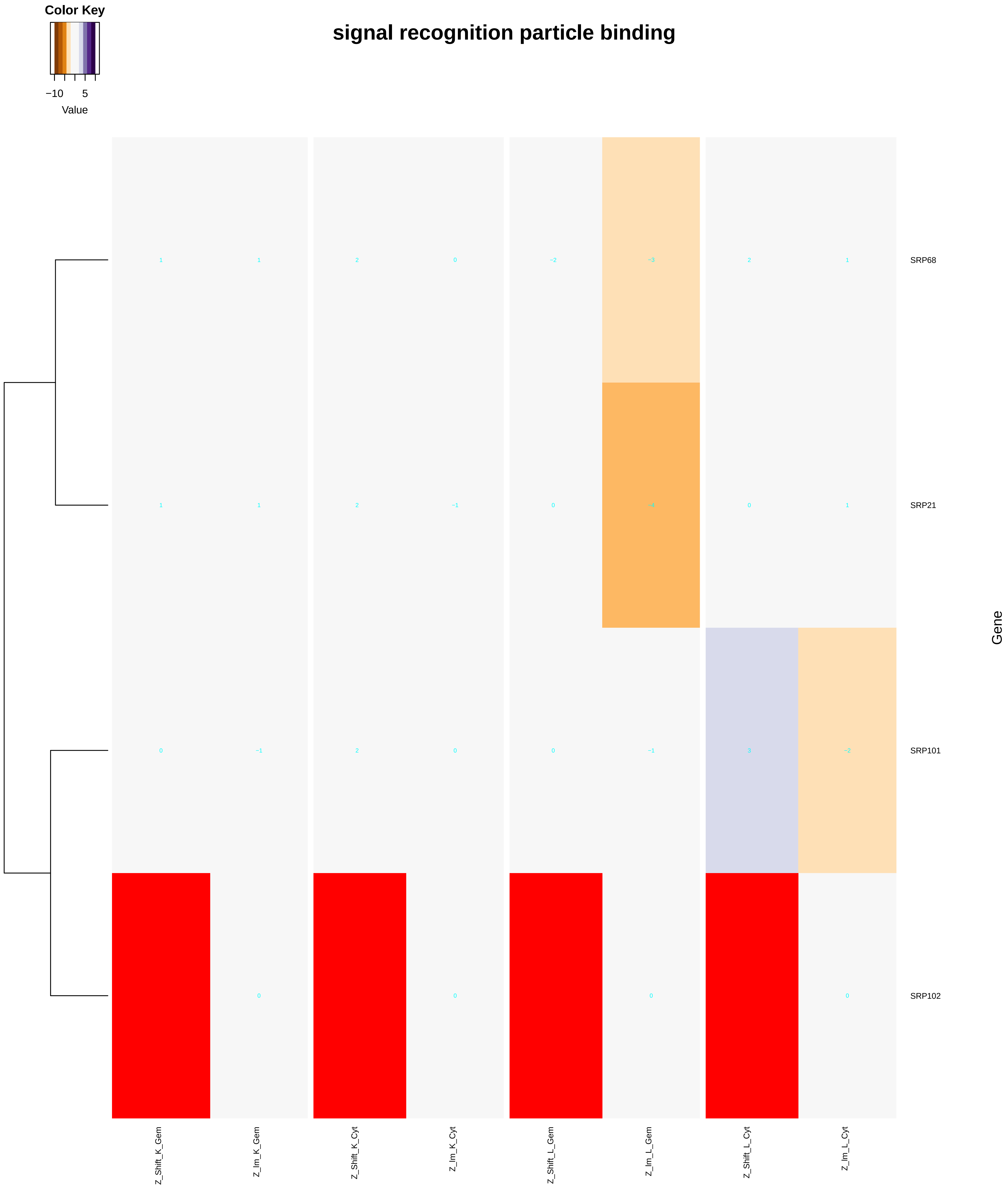
signal recognition particle binding
Color Key
−10
5
Value
SRP68
SRP21
SRP101
SRP102
Gene
Z_Shift_K_Gem
Z_Im_K_Gem
Z_Shift_K_Cyt
Z_Im_K_Cyt
Z_Shift_L_Gem
Z_Im_L_Gem
Z_Shift_L_Cyt
Z_Im_L_Cyt

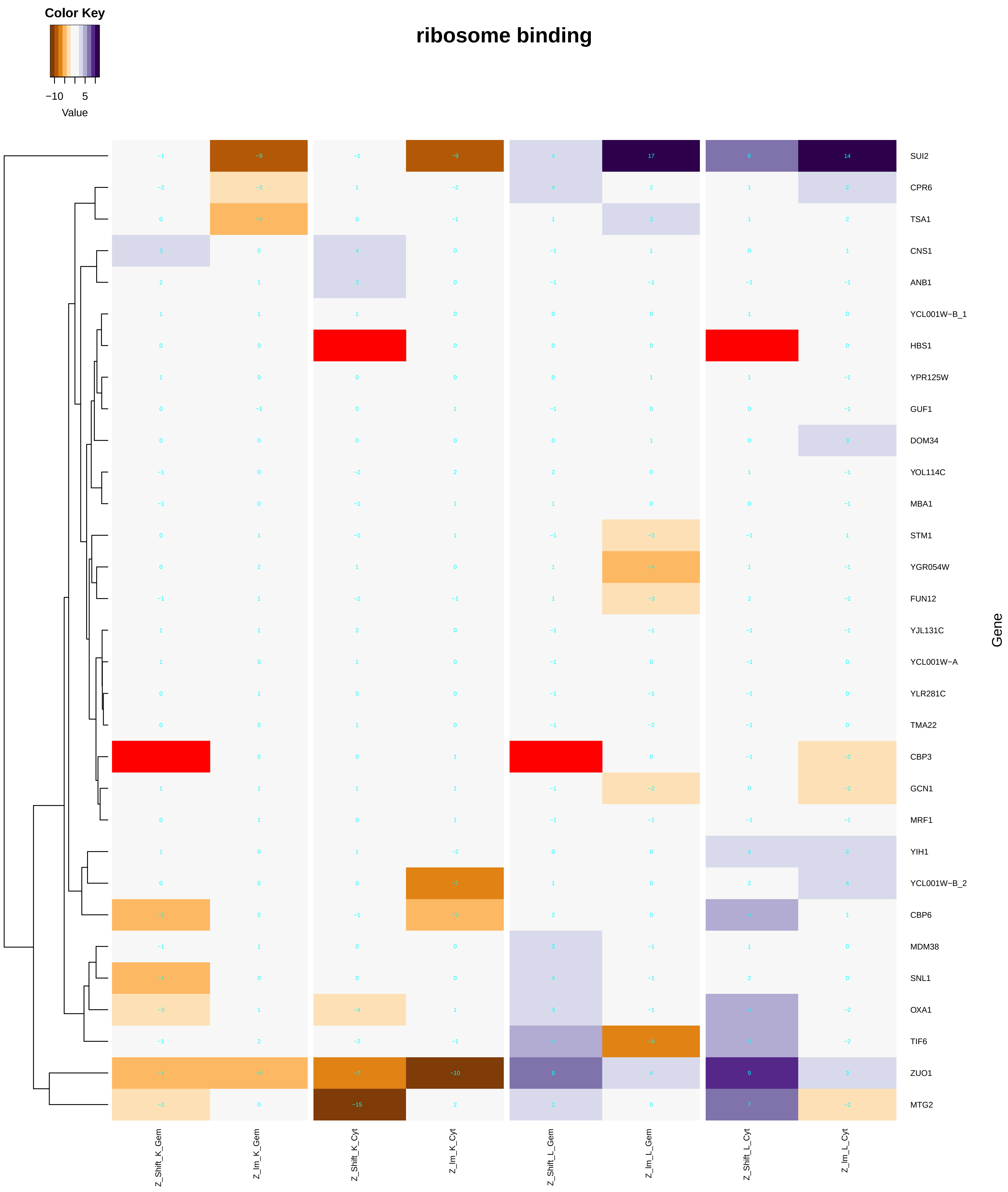

ribosome binding
Color Key
−10
5
Value
SUI2
CPR6
TSA1
CNS1
ANB1
YCL001W−B_1
HBS1
YPR125W
GUF1
DOM34
YOL114C
MBA1
STM1
YGR054W
FUN12
YJL131C
YCL001W−A
YLR281C
TMA22
CBP3
GCN1
MRF1
YIH1
YCL001W−B_2
CBP6
MDM38
SNL1
OXA1
TIF6
ZUO1
MTG2
Gene
Z_Shift_K_Gem
Z_Im_K_Gem
Z_Shift_K_Cyt
Z_Im_K_Cyt
Z_Shift_L_Gem
Z_Im_L_Gem
Z_Shift_L_Cyt
Z_Im_L_Cyt

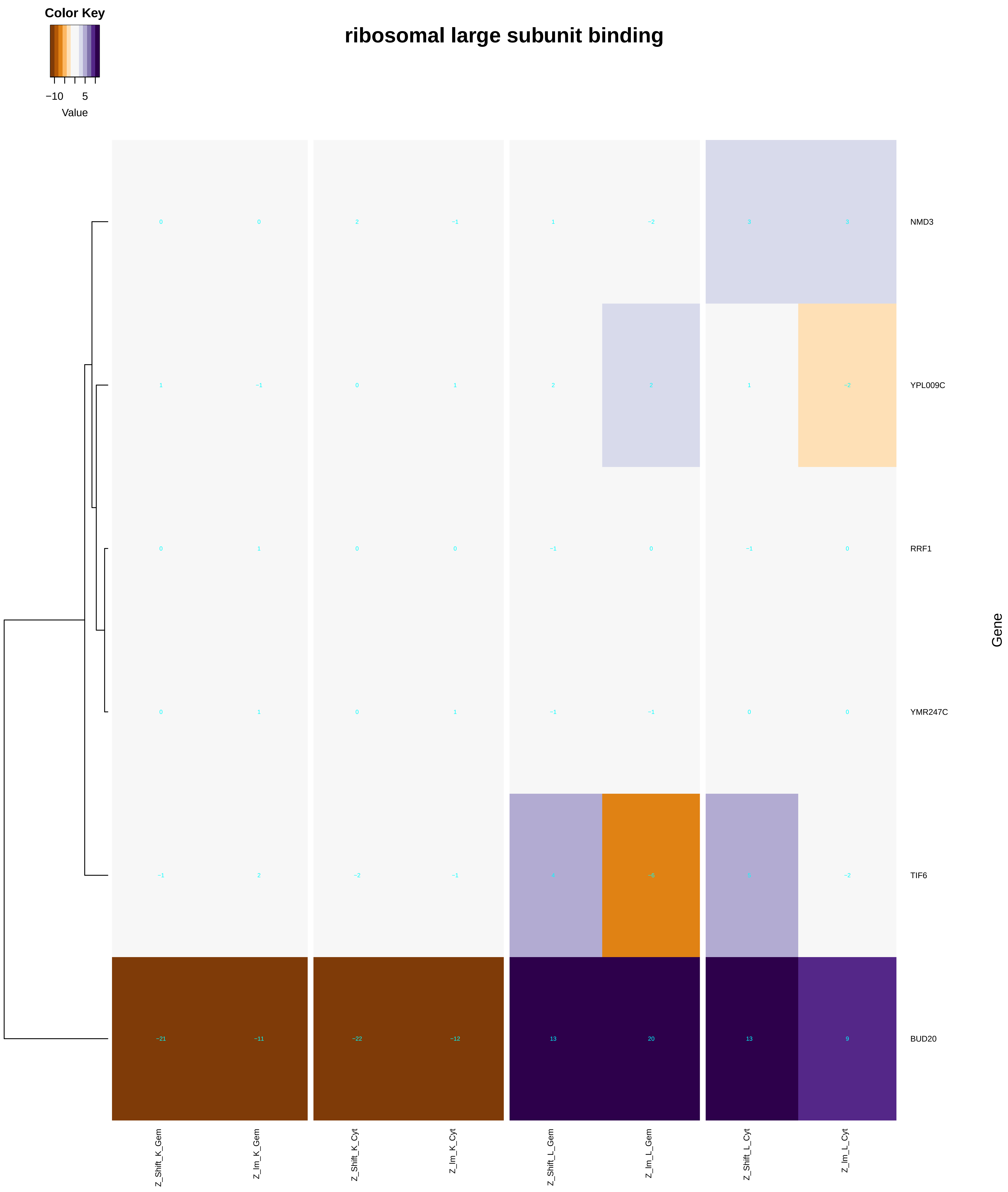

Color Key
−10
5
Value
ribosomal large subunit binding
NMD3
YPL009C
RRF1
YMR247C
TIF6
BUD20
Gene
Z_Shift_K_Gem
Z_Im_K_Gem
Z_Shift_K_Cyt
Z_Im_K_Cyt
Z_Shift_L_Gem
Z_Im_L_Gem
Z_Shift_L_Cyt
Z_Im_L_Cyt

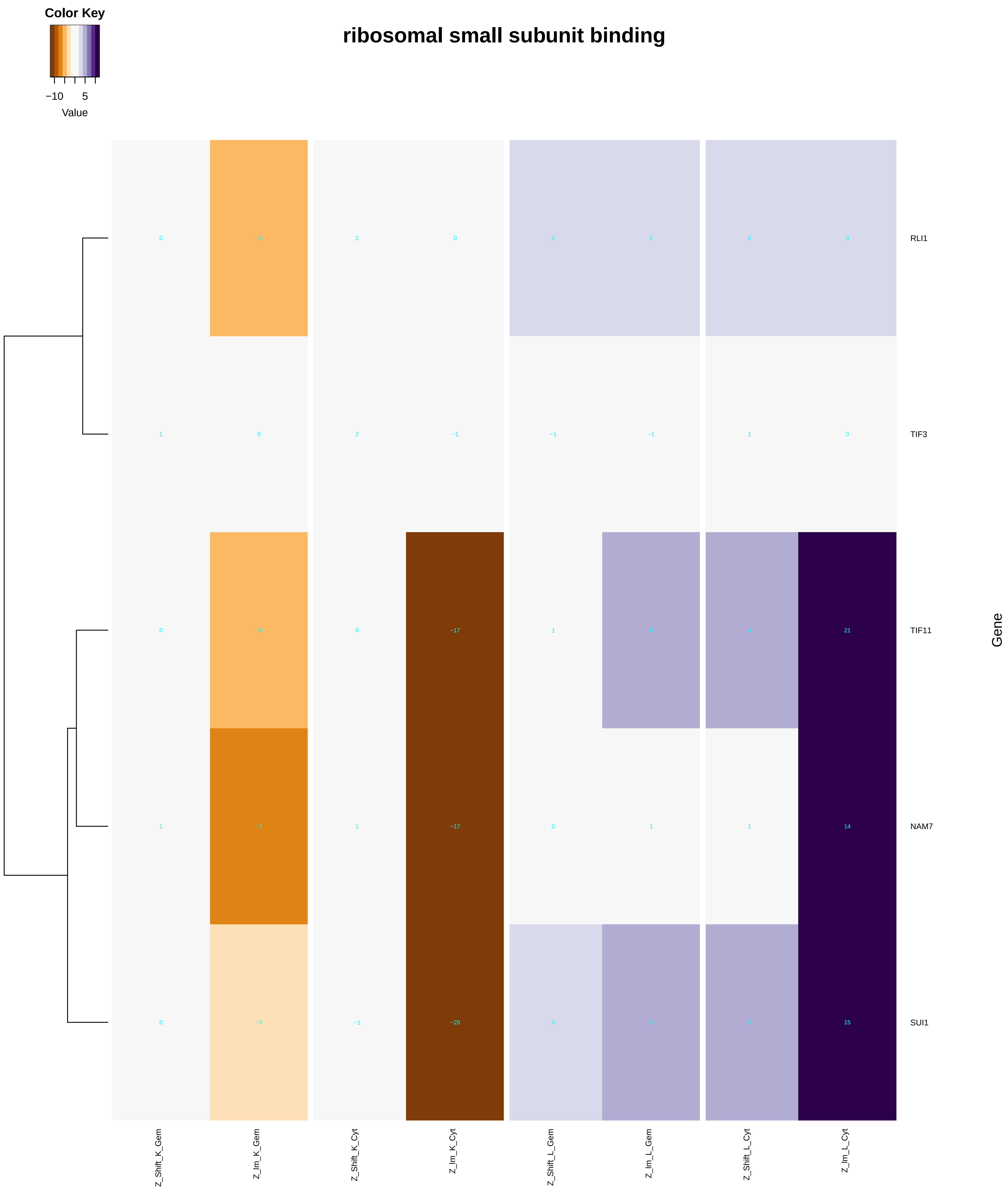

ribosomal small subunit binding
Color Key
−10
5
Value
0
−5
2
0
2
2
3
3
RLI1
1
0
2
−1
−1
−1
1
0
TIF3
0
−6
0
−17
1
21
TIF11
1
−7
1
−17
0
1
1
14
NAM7
0
−3
−1
−26
3
15
SUI1
Gene
Z_Shift_K_Gem
Z_Im_K_Gem
Z_Shift_K_Cyt
Z_Im_K_Cyt
Z_Shift_L_Gem
Z_Im_L_Gem
Z_Shift_L_Cyt
Z_Im_L_Cyt
